# Different Formulations of Levothyroxine for Treating Hypothyroidism: A Real-Life Study

**DOI:** 10.1155/2020/4524759

**Published:** 2020-01-20

**Authors:** Pierpaolo Trimboli, Lorenzo Scappaticcio, Annamaria De Bellis, Maria Ida Maiorino, Luisa Knappe, Katherine Esposito, Giuseppe Bellastella, Luca Giovanella

**Affiliations:** ^1^Clinic for Nuclear Medicine and Competence Centre for Thyroid Disease, Imaging Institute of Southern Switzerland, Ente Ospedaliero Cantonale, Bellinzona, Switzerland; ^2^Unit of Endocrinology and Metabolic Diseases, University of Campania “L. Vanvitelli”, Naples, Italy; ^3^Department of Advanced Medical and Surgical Sciences, University of Campania “L. Vanvitelli”, Naples, Italy; ^4^Clinic for Nuclear Medicine, University Hospital and University of Zurich, Zurich, Switzerland

## Abstract

**Objective:**

Hypothyroid patients are treated by sodium levothyroxine (LT4). Tablet is the mostly used LT4 formulation, and the fasting regimen is required for the absorption of active principle. Also, gastrointestinal diseases and drugs may impair the LT4 bioavailability when tablet is used. Nonsolid LT4 formulations (i.e., liquid solution (LS) and soft gel (SG) capsule) were manufactured to overcome the limitations of LT4 tablet. This study was conceived to evaluate the performance of nonsolid LT4 formulations in a real-life scenario.

**Methods:**

Two institutions participated in the study that was conducted in two phases (i.e., enrollment and re-evaluation). Adults with autoimmune or postsurgical hypothyroidism and on LT4 from a few months were selected. A nonparametric statistical analysis for paired or unpaired data was performed.

**Results:**

121 consecutive cases were included. At the enrollment phase, a 52% of patients took the therapy at least 30 min before breakfast with no difference between tablet and SG/LS users. TSH was 1.65 mIU/L (0.86–2.70) in patients on LT4 tablet and 1.70 mIU/L (1.10–2.17) in those on SG/LS (*p*=0.66). At the re-evaluation phase, among the patients using correct LT4 assumption, the TSH value was stable in the tablet group (*p*=0.66). At the re-evaluation phase, among the patients using correct LT4 assumption, the TSH value was stable in the tablet group (*p*=0.66). At the re-evaluation phase, among the patients using correct LT4 assumption, the TSH value was stable in the tablet group (*p*=0.66). At the re-evaluation phase, among the patients using correct LT4 assumption, the TSH value was stable in the tablet group (*p*=0.66). At the re-evaluation phase, among the patients using correct LT4 assumption, the TSH value was stable in the tablet group (

**Conclusion:**

The performance of nonsolid LT4 formulations is not influenced by correct or incorrect use of therapy. On the contrary, LT4 tablet does not guarantee euthyroidism when it is ingested without waiting for at least 30 minutes before breakfast. These new data, obtained in a real-life scenario, suggest that LT4 SG/LS should be regarded as first-line therapy for treating adults with newly diagnosed hypothyroidism.

## 1. Introduction

Hypothyroid patients are electively treated by sodium levothyroxine (LT4) [1]. LT4 tablet is the most widely used formulation, which is taken per os and disintegrated and dissolved in the stomach [2]. These events are essential preconditions for the effective drug absorption which takes place in the small intestine [3]. The fasting regimen is required to promote the absorption of LT4 tablet and its ingestion 30 minutes before breakfast is necessary [1]. It is well known that in addition to the coadministration with foods and beverages, gastrointestinal diseases (i.e., *Helicobacter pylori* gastritis, atrophic gastritis, celiac disease, lactose malabsorption/intolerance, and dysbiosis), and drugs (i.e., proton-pump inhibitors, aluminum-containing antacids, calcium carbonate, ferrous sulphate, sucralfate, raloxifene, bile acid sequestrants, and phosphate binders) may substantially impair the bioavailability of LT4 tablet preparation [4]. New LT4 formulations, namely, liquid solution (LS) and soft gel (SG) capsule, were manufactured to overcome the limitations of the tablet [4, 5]. LS preparation, in which LT4 is dissolved in ethanol and glycerol, has proven to be bioequivalent to tablet [6]. SG is the gelled form of LS in which LT4 is dissolved in water and glycerin; it showed a 50% reduction 10 min after the ingestion and a complete disappearance 11 min later [6]. Since the LS and SG commercialization, a growing number of studies reported their advantages [7–13]. Most of these studies evaluated the changes of TSH attained by switching from tablet to LS or SG in selected patients consistently maintaining the prescribed LT4 regimen (i.e., such as 30 minutes before breakfast), thus introducing a performance bias [9, 11, 12]. Moreover, in some studies [7, 10], there might be a selection bias for having included patients with potentially impaired absorption of LT4 tablet along with a TSH not on target. Yet, since authors and editors are usually ready to publish enthusiastic findings for a new drug, a publication and reporting bias could not be excluded in this field. However, at the same time, thyroidologists are keenly aware of how in clinical practice it may not be easy to keep the TSH therapeutic target since patients might not follow the initial instructions for a correct use of LT4 or they might change administration regimen over time.

For the above reasons, here, we conceived a study aiming to assess the performance of LT4 LS/SG in a cohort of hypothyroid patients without causes of potential malabsorption of LT4 tablet and proving recent restored euthyroidism. This setting allowed us to evaluate LT4 performance in a real-life scenario, reducing or deleting the biases otherwise present in other papers. The outcomes of the present study were to (1) investigate how hypothyroid patients with proven restored euthyroidism really follow the initial instructions for the correct use of LT4 and (2) analyze the performance of LT4 LS/SG versus tablet in the different administration regimens of patients.

## 2. Methods

### 2.1. Study Design

Two institutions participated to this study: Thyroid Centre of Ente Ospedaliero Cantonale in Lugano/Bellinzona (Switzerland) and Unit of Endocrinology and Metabolic Diseases of University of Campania “L. Vanvitelliˮ in Naples (Italy). The study design was conducted in two phases.

The enrollment phase was from March to July 2018. At this step, consecutive hypothyroid patients were screened for their eligibility. As the major inclusion criterion, we considered the adults with hypothyroidism due to Hashimoto's thyroiditis or thyroidectomy for benign goiter who were on LT4 therapy for no more than six months. As further inclusion criteria, we had patients (1) having normal TSH (see below) both previously and at last visit performed at our institutions, (2) using any commercialized LT4 preparations with unchanged dose over time, and (3) having stable body weight during the last six months. Excluded were patients (1) proven to have or suspected for intestinal malabsorption, (2) using drugs with potential interference on LT4 absorption, (3) with cardiovascular symptoms (i.e., arrhythmia and atrial fibrillation), (4) with heart, renal, and/or hepatic failure, (5) with recent or current infection/inflammation, and (6) in pregnancy. Following data were recorded in all included patients: hour of LT4 ingestion, hour of breakfast, and time interval between LT4 ingestion and breakfast. Considering that for all patients the restored euthyroidism on LT4 therapy was demonstrated in two follow-up visits, the same therapy (i.e., LT4 formulation and doses) was maintained, and a 6-month control visit was planned.

**Figure 1:**
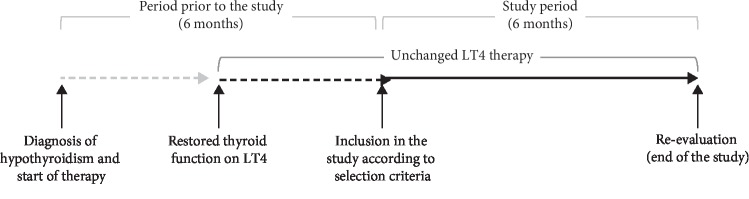
Schematic representation of the path of each patient included in the study. Diagnosis of hypothyroidism could not be posed at our institutions. The grey line indicates that patients could be followed-up not at our institutions. Continuous and dashed black lines indicate follow-up at our institutions. Selection criteria for the study are reported in the text.

At the re-evaluation phase, patients were re-examined as usual. At this point, patients were excluded from the study if they (1) changed LT4 formulation and/or dose, (2) experienced a modification of the body weight of at least two kilograms, (3) had recent infections/inflammation, and (4) reported clinical news (i.e., development of gastrointestinal symptoms or diseases, assumption of drugs impairing L-T4 absorption, and pregnancy).  Figure 1 illustrates schematically the path of each patient included in the study.

### 2.2. Levothyroxine Formulations

The patients from Italy used LT4 tablet commercialized by Merck Serono S.p.A. (Via Casilina 125, 00176 Roma, Italia). LT4 tablet used in Switzerland was commercialized by Merck AG (Chamerstrasse 174, 6300 Zug, Switzerland). Soft gel and liquid solution were commercialized by IBSA Institut Biochimique SA (Via del Piano 29, 6926 Collina d'Oro, Switzerland).

### 2.3. Laboratory Measurement

In both the institutions participating to the study, thyroid function was assessed by an automated immunoassay method (Elecsys® e601 Roche Diagnostics). This assay had analytical sensitivity for TSH of 0.006 mIU/L, with reference range between 0.30 and 4.20 mIU/L. Serum levels of free-T4 (ranging from 12 to 22 pmol/L), free-T3 (3.1 to 6.8 pmol/L) were automatically measured only in presence of a skewed TSH value. Baseline diagnostic thyroid results were extracted for the present analysis.

### 2.4. Statistical Analysis

In the present paper, continuous parameters were expressed as median and interquartile ranges (IQR). A nonparametric statistical analysis for paired or unpaired data was performed. Frequencies were compared by the Chi-squared test. Statistical significance was set at *p* < 0.05. Statistical analysis was performed by GraphPad version 7 (GraphPad Prism, La Jolla CA, USA).

## 3. Results

According to our selection criteria, a cohort of 133 patients were initially enrolled, and all the patients completed the study period. Among these cases, 12 were excluded because they changed LT4 formulation or dose (*n* = 9) and had modification of body weight (*n* = 2) or had recent infection (*n* = 1). The final study series included 121 patients, 53 from Lugano/Bellinzona, and 68 from Naples. Two-thirds of patients used tablet and one-third SG or LS.  Table 1 details the main characteristics of the study series.

**Figure 2:**
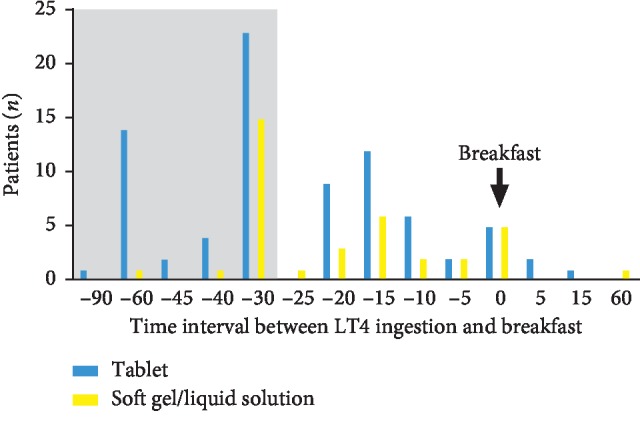
Distribution of patients according to the regimen of LT4 ingestion with respect to the time of breakfast. The grey zone represents the optimal time of LT4 ingestion with respect to the time of breakfast (i.e., at least 30 min before). Three cases of the series varied the time of LT4 ingestion and breakfast, and they are not reported in this figure.

At baseline (i.e., enrollment phase), the median TSH in the overall cohort was 1.70 mIU/L, being 1.65 mIU/L (0.86–2.70) in patients on LT4 tablet and 1.70 mIU/L (1.10–2.17) in those on SG/LS (*p*=0.66). Median daily LT4 dose was 100 *μ*g per patient and 1.33 *μ*g per body kg, with no difference according LT4 preparations. No significant difference was found between Swiss and Italian patients in gender, age, body weight, TSH, and LT4 dose. The relationship between daily LT4 therapy and breakfast was investigated. Overall, a 52% of patients took the therapy at least 30 min before breakfast; this percentage was not significantly different between tablet and SG/LS users (44/52.3% and 17/45.9%, respectively).  Figure 2 shows the distribution of patients according to the time interval between LT4 ingestion and breakfast.

At the re-evaluation phase, all the patients confirmed their daily regimen of LT4 use (i.e., unchanged dose and modality of ingestion), and the overall median TSH was 1.60 mIU/L. TSH value was significantly lower in the group of patients on LT4 SG/LS than those on tablet (Table 2). This finding was confirmed also when each group was split according to the correct use of LT4 (i.e., at least 30 min before breakfast) or not (Table 2). There were nine cases with elevated TSH (>4.20 mIU/L), eight (9.52%) on LT4 tablet, and one (2.70%) on SG/LS.

**Figure 3:**
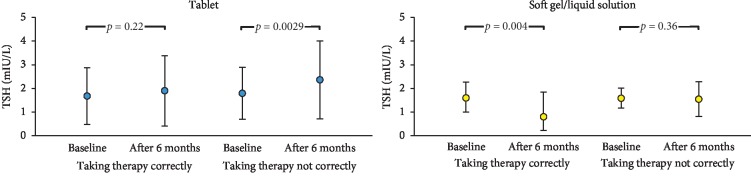
Analysis of TSH values recorded in the groups of patients on LT4 tablet or SG/LS. TSH is reported for each subgroup as median (and IQR). No significant difference was recorded among the subgroups relative to age, body weight, LT4 dose, and hypothyroidism cause.

The baseline and final TSH value were evaluated according to the LT4 formulation. Among the patients using LT4 correctly, TSH value was stable in tablet group and significantly reduced TSH in the SG/LS group. Among the patients using therapy not correctly, TSH was significantly increased in those on tablet, while no difference of TSH was observed in those on SG/LS (Figure 3).

## 4. Discussion

The present observational study was conceived to evaluate the performance of LT4 nonsolid formulations in a real-life scenario. With this purpose, two thyroid institutions working in two countries participated and enrolled hypothyroid patients without intestinal malabsorption and using any commercialized LT4 formulations. It has to be underlined that while LT4 tablet has been used in both Switzerland and Italy since 80s, SG was introduced in both countries about ten years ago, and LS has been commercialized only in Italy. Also, to be noticed, Italian hypothyroid patients are charged of costs for LT4 SG, while there are no costs to use tablet. Therefore, we can affirm that the two institutions have worked in two different socio-economic environments. At baseline, all our patients had restored euthyroidism even if a half of them was taking LT4 at least 30 min before breakfast. To avoid a performance bias, the patients were asked to continue their LT4 therapy. At the end of the study, the group of patients taking LT4 SG/LS had a significantly lower TSH than the tablet group, even after assessing on the basis of correct/incorrect use of the therapy (Table 2). Furthermore, in the SG/LS group, TSH values remained stable when therapy was incorrectly taken and lowered when therapy was correctly followed. On the contrary, patients on LT4 tablet had a significant increase of TSH levels and nine percent of them lost the euthyroid state following a not correct use of therapy (Figure 2).

According to the American Thyroid Association guidelines, LT4 LS and SG may be considered in the rare case of putative allergies to excipients [1]. Moreover, no recommendations are reported by other specialized societies about the indications for the use of these novel LT4 formulations [14–16]. Giving the high level of evidence on the efficacy and safety of LT4 LS/SG formulations [9, 12, 13] subsequent to these guidelines [1, 14–16] and considering the worldwide prevalence of gastrointestinal disorders that considerably impact on LT4 tablet absorption [17, 18], the indications of LT4 nontablet formulations should be reappraised.

Here, we enrolled hypothyroid patients with no apparent gastrointestinal malabsorption who were not using drugs with potential of interfere on LT4 tablet absorption. The reliability of LT4 SG/LS had already been evaluated in this scenario by two studies having different design [9, 11]. In one study [9], 152 cases were switched from LT4 tablet to LS and TSH decreased at 1–3 months with further reduction at 5–7 months. In the other one [11], patients with or without well-restored euthyroidism on LT4 tablet were switched to SG with TSH lowering after three months. In both these studies [9, 11], patients were asked to take LT4 therapy at least 30 min before breakfast. Fallahi et al. [9] stated that the better results of LT4 LS were due to its pharmacokinetic profile [19–21], while they did not give explanations of the further decrease of TSH over time. In the present study, we observed similar trend with declining levels of TSH, and some speculations should be done to explain these findings. First, here, we collected data at baseline and after six months. Thus, we had no intermediate data, and we cannot exclude that the 6-month result might have been present already earlier. Second, we may assume that the patients taking correctly LT4 could be considered more adherent than those who did not correctly ingest the therapy. Third, the easier patient compliance with LT4 SG/LS [8, 17] could explain the lowering trend in TSH levels with the new formulations. To date, no studies exist with follow-up longer than that reported here and by Fallahi et al. [9], and further pharmacokinetics studies are advised. Moreover, on the contrary, our findings of patients on LT4 tablet are relevant, considering the risk of increased TSH if patients do not correctly take the therapy. Finally, it has to be underlined that we did not include patients using other therapies which interfere on LT4 tablet absorption. Then, our data show that nonsolid LT4 formulations provide a better absorption of levothyroxine than the tablet preparation also in patients without interfering factors.

Strengths and limitations of the present study should be discussed. Here, we aimed to enroll hypothyroid patients with restored euthyroidism on LT4 tablet, SG or LS formulations and we did not influence their prescribed regimen. This allowed us to avoid a performance bias (i.e., the bias present if investigators and/or patients are influenced by the study design). Also, no patients were lost during follow-up so that these data were not affected by attrition bias. On the contrary, here, we included only patients without putative malabsorption so that the present results could be considered as reliable only in this setting. Yet, the follow-up time of our patients may be considered too short, and the findings might be different later. Lastly, our results mostly applied to LT4 SG versus tablet since a few patients used LS formulation. This has to be considered as a major limitation about the LS. In fact, the liquid LT4 commercialized in Italy during the study period contained alcohol, and this might reduce the patient's compliance. Because only three cases were taking LS, our study did not furnish sufficient data on this formulation.

## 5. Conclusion

The present study was undertaken to evaluate the performance of LT4 nonsolid preparations in hypothyroid patients with proved restored euthyroidism regardless of whether they used LT4 SG/LS or tablet. A half of the patients used LT4 without waiting for at least 30 minutes before breakfast. This fact became relevant only for tablet users, while the performance of LT4 LS and SG was not impaired by the incorrect use. These new data, obtained in a real-life scenario, suggest that LT4 SG/LS should be regarded as first-line therapy for treating adult subjects with newly diagnosed hypothyroidism.

## Figures and Tables

**Table 1:** Main features of study series.

Demographic parameters	
Female/male	101/20
Age (year)	51 (42–62)
Body weight (kg)	70 (62–83)

LT4 formulation	
Tablet	84 (69.4%)
Soft gel	34 (28.1%)
Liquid solution	3 (2.5%)

Age and weight are reported as median (and IQR); used formulations are reported as number of patients (and percentage).

**Table 2:** Results of the TSH values recorded at the end of the study.

	LT4 Tablet	LT4 soft gel/liquid solution	*p*
All patients	1.97 (0.70–3.45)	1.15 (0.30–1.84)	0.0033
Patients taking therapy at least 30 min before breakfast	1.67 (0.51–3.07)	0.80 (0.22–1.84)	0.0318
Patients taking therapy not correctly	2.30 (0.75–3.78)	1.24 (0.35–1.95)	0.0400

All TSH values are reported as median (and IQR).

## Data Availability

The data used to support the findings of this study are available from the corresponding author upon request.
